# Hybrid Acrylated Chitosan and Thiolated Pectin Cross-Linked Hydrogels with Tunable Properties

**DOI:** 10.3390/polym13020266

**Published:** 2021-01-14

**Authors:** Shaked Eliyahu, Alexandra Galitsky, Esther Ritov, Havazelet Bianco-Peled

**Affiliations:** 1 The Russell Berrie Nanotechnology Institute, Technion-Israel Institute of Technology, Haifa 3200003, Israel; shaked@campus.technion.ac.il; 2 Department of Chemical Engineering, Technion-Israel Institute of Technology, Haifa 3200003, Israel; gsashaaa@gmail.com (A.G.); esty.ritov@gmail.com (E.R.)

**Keywords:** acrylated chitosan, thiolated pectin, hybrid hydrogels, mucosal mimetic, polysaccharide hydrogels

## Abstract

We developed and characterized a new hydrogel system based on the physical and chemical interactions of pectin partially modified with thiol groups and chitosan modified with acrylate end groups. Gelation occurred at high pectin thiol ratios, indicating that a low acrylated chitosan concentration in the hydrogel had a profound effect on the cross-linking. Turbidity, Fourier transform infrared spectroscopy, and free thiol determination analyses were performed to determine the relationships of the different bonds inside the gel. At low pH values below the pKa of chitosan, more electrostatic interactions were formed between opposite charges, but at high pH values, the Michael-type addition reaction between acrylate and thiol took place, creating harder hydrogels. Swelling experiments and Young’s modulus measurements were performed to study the structure and properties of the resultant hydrogels. The nanostructure was examined using small-angle X-ray scattering. The texture profile analysis showed a unique property of hydrogel adhesiveness. By implementing changes in the preparation procedure, we controlled the hydrogel properties. This hybrid hydrogel system can be a good candidate for a wide range of biomedical applications, such as a mucosal biomimetic surface for mucoadhesive testing.

## 1. Introduction

Polysaccharide-based hydrogels are three-dimensional structured networks with great water absorbance ability. As they are biodegradable and biocompatible, they are utilized for a wide range of biomedical applications, such as regenerative medicine and sustained drug release systems [1,2,3,4].

Chitosan is a cationic biocompatible polysaccharide derived from the deacetylation of the acetyl group in chitin and is soluble below its pKa of 6.2–7.0 [5]. It has emerged as a promising polymer for biomedicine applications, including nanosystems, films, and hydrogels [6,7]. Pectin is an anionic polysaccharide extracted from plants’ middle lamella and primary cell walls. This polymer is mainly utilized as a thickening and gelling agent in the food industry, but it is also widely studied for drug delivery systems in the pharmaceutical field. The pKa of pectin is around 2.9–3.2 [8]. A mixture of pectin and chitosan has been found to form stable hydrogels [9]. Such hydrogels can be fabricated by mixing the hot acidic solutions of the polymers where the cross-linking occurs due to hydrogen bonding formation upon cooling [10]. Pectin chitosan thermo-reversible hydrogels have been examined for pharmaceutical applications, but their acidic pH limits their range of use [8]. Alternatively, hydrogels based on a polyelectrolyte complex between pectin and chitosan are formed by establishing strong and attractive interactions in a wider pH range [11]. However, it was pointed out by Bernabé and coworkers that chitosan-pectin complex membranes would be totally destroyed at extreme pH values [12].

Therefore, the incorporation of another cross-linking method can improve certain properties, such as swelling ability, gel strength, and thermal stability. Chemical cross-linking has the advantages of endurance and stability, but it typically requires the presence of UV light and/or a chemical precursor, which is usually cytotoxic [13]. To overcome these limitations, the Michael-type addition reaction can be implemented. This reaction is typically free of byproducts and can occur without catalysts because of its mechanism [14,15,16,17].

In this work, we developed and characterized a new one-pot hydrogel system based on the physical and chemical interactions of acrylated chitosan and thiolated pectin. Acrylated chitosan was previously synthesized by grafting poly (ethylene glycol) diacrylate (PEGDA) chains on a chitosan backbone and was evaluated as a mucoadhesive polymer by Shitrit et al. [18]. Thiolated pectin is produced by conjugating pectin with molecules carrying free thiol functionality, usually cysteines [19]. In this hybrid system, physical interactions are formed between the positively charged amine on the chitosan backbone and the negatively charged carboxylic acid in pectin. The chemical reaction is mediated using the Michael-type addition reaction between thiol and acrylate. This dual cross-linking enabled hydrogel preparation in various conditions. We analyzed the gelation process in different conditions to investigate the involvement of the different interactions: above the pKa of pectin and below the pKa of chitosan in two pH values and above the pKa of pectin and chitosan. The hydrogels were evaluated using Fourier transform infrared spectroscopy (FTIR), turbidity measurements, and free thiol determination. The mechanical properties, swelling, and hydrogel adhesion were characterized. To the best of our knowledge, this is the first time such hydrogels have been described.

## 2. Materials and Methods

### 2.1. Materials

Low-molecular-weight chitosan (molecular weight of 207 kDa, deacetylation degree of 77.6%), 5,5′-dithiobis(2-nitro-benzoic acid) (Ellman’s reagent), and fluorescamine were obtained from Sigma Aldrich (Rehovot, Israel). Sodium tripolyphosphate was purchased from Alfa Aesar (Lancashire, UK). Sodium chloride and NaOH were obtained from Bio-Lab Ltd. (Jerusalem, Israel). Acetic acid glacial, dimethyl sulfoxide (DMSO), Na_2_HPO_4_, NaH_2_PO_4_ * H_2_O, KH_2_PO_4_, sodium acetate and L-cysteine monohydrate hydrochloride were purchased from Merck (Darmstadt, Germany). Potassium chloride was obtained from Nile Chemicals (Mumbai, India). PEGDA with a molecular weight of 10 kDa was obtained from the laboratory of Biomaterials and Regenerative Medicine at the Department of Biomedical Engineering, Technion, Israel. 1-Ethyl-3-(3-dimethylaminopropyl)-carbodiimide hydrochloride (EDAC) was purchased from Tzamal D-Chem (Petach Tikva, Israel). Classic citrus pectin (CU 701; degree of esterification of 34%, GalA of 86%) was kindly donated by Herbstreith & Fox (Neuenbürg, Germany).

### 2.2. Buffer Preparation

#### 2.2.1. Acetate Buffer

To prepare the acetate buffer, acetic acid was dissolved in double distilled water (DDW) to a final concentration of 0.1 M, while a powder of sodium acetate was dissolved in DDW to the same final concentration of 0.1 M. To obtain a buffer solution with a pH value of 4, 847 mL of the acetic acid solution was mixed with 153 mL of the sodium acetate solution, and the pH value was measured and adjusted to 4 using 1 M NaOH solution. To obtain a pH value of 5.6, 95 mL of the acetic acid solution was mixed with 905 mL of the sodium acetate solution. The pH value was measured and adjusted to 5.6.

#### 2.2.2. Phosphate Buffer Saline (PBS)

PBS was prepared by dissolving 137 mM NaCl, 2.7 mM KCl, 10 mM Na_2_HPO_4_, and 1.8 mM KH_2_PO_4_ in DDW. The pH value was measured and adjusted to either 7.4 or 6.5 using a 1 M HCl solution.

#### 2.2.3. Phosphate Buffer (PB)

Phosphate buffer solution of 0.1 M was prepared using 3.1 g of NaH_2_PO_4_ * H_2_O and 10.9 g of Na_2_HPO_4_, which were dissolved in 1 L of DDW. Following complete dissolving, the pH was adjusted to 8 using a 5 M NaOH solution.

### 2.3. Synthesis of Acrylated Chitosan

Acrylated chitosan was synthesized as previously described [18]. Chitosan (1 g) was dissolved in 100 mL of 2% (*v/v*) acetic acid overnight at room temperature, followed by an addition of 1 g of PEGDA and stirring for 15 min. The reaction mixture was incubated for 3 h in the dark under shaking at a speed of 100 rpm at 60 °C. The mixture was dialyzed in the dark using a dialysis bag, with a molecular weight cut-off of 12–14 kDa against 5 L of DDW for three days. After dialysis, the product was filtered with a Buchner funnel, frozen, lyophilized at 0.01 mbar and −30 °C, and stored at −20 °C until further use.

### 2.4. Synthesis of Thiolated Pectin

Thiolated pectin was synthesized using the primary amine groups of the amino acid cysteine, which was covalently anchored to the carboxylic acid groups of pectin. The synthesis was performed according to the procedure previously reported by Majzoob et al. [19] with some modifications.

Pectin of 1 g was dissolved in 100 mL of DDW and stirred overnight to form a homogeneous solution. EDAC was added to a final concentration of 50 mM, activating the pectin’s carboxylic acid groups. The pH was adjusted to 4.75 using 1 M NaOH solution, and the reaction was allowed to proceed for 1 h. Then, 2 g of L-cysteine monohydrate hydrochloride was dissolved in DDW and added at a weight ratio of 2:1 (pectin: cysteine). The pH was adjusted to 5, and the mixture was incubated for 24 h in the dark at room temperature under stirring. The resultant conjugated pectin-cysteine was isolated by dialysis at room temperature in the dark using a cellulose membrane with a 12–14 kDa molecular weight cut-off against 1 mM HCl, twice against 1 mM HCl containing 1% NaCl, and finally, once against 1 mM HCl. The polymer solution was frozen, and the material was lyophilized at −30 °C and 0.01 mbar and stored at 4 °C until further use.

The amount of free thiol groups was determined using Ellman’s reagent reaction according to the method described in Section 2.8 and was found to be 0.35 mM.

### 2.5. Hydrogel Fabrication

Thiolated pectin and acrylated chitosan hydrogels were prepared by mixing the two polymer solutions at different pH values. A solution of thiolated pectin (1.33% (*w*/*v*)) was dissolved in buffer and stirred overnight. A solution of acrylated chitosan (1% (*w*/*v*)) was dissolved separately in the same buffer and stirred overnight. Subsequently, the two solutions were mixed in pre-determined ratios of thiol to acrylate (5:1, 3:1, 1:1, 1:3, 1:5) using a vortex and casted into a cylinder mold (14 mm in diameter and 4 mm in height) made of Teflon. Gelation was allowed to proceed for different time periods at room temperature in humid conditions.

Gelation was estimated using the vial tilting method, as reported earlier, by flipping the vial and determining whether the mixture flows [12]. A mixture was considered to be in the gel state if there was no flow within 1 min. At the end of the time noted, the vial was tilted again. The mixture was classified as a soft gel if the gel broke and started to flow as a result of the second tilt. The experiments were performed in triplicate.

### 2.6. Turbidity Measurements

Turbidity measurements were performed to indicate the extent of electrostatic interactions between the primary amine of acrylated chitosan and the carboxylic acid in thiolated pectin. Thiolated pectin and acrylated chitosan were separately dissolved in different pH values, as described in Section 2.5, and mixed. Before curing, the solutions were poured into a 96-well plate and left to cure at room temperature. The absorbance was measured using a Synergy™ HTBioTek^®^ (BioTek Instruments, Winooski, VT, USA) at a wavelength of 380 nm. All samples were compared with the absorbance of thiolated pectin solutions mixed with buffer instead of acrylated chitosan solutions.

### 2.7. FTIR

FTIR spectra were recorded using a Nicolet 6700 FTIR (ThermoScientific, Waltham, MA, USA) coupled to a liquid nitrogen-cooled mercury–cadmium–telluride (MCT) detector in ATR mode. The hydrogel samples were freeze-dried at 0.01 mbar and −30 °C and ground to a powder. The spectra were at an average of 128 scans at a resolution of 4 cm^−1^, and they were corrected for the baseline and smoothed.

### 2.8. Free Thiol Group Determination

The amount of free unreacted thiol groups in the resultant hydrogels was determined using Ellman’s reagent reaction, as described by Eshel-Green et al. with modifications [20]. The hydrogels were prepared and casted as described in Section 2.5. Then, the unreacted polymer chains were extracted by submerging the hydrogels in 1.5 mL DDW for 30 min in the dark. The tubes were centrifuged using a Megafuge 1.0 centrifuge (Heraeus, Hanau, Germany) at 3300 g for 5 min. Then, 250 µL of the upper liquid containing the unreacted chains was mixed with 2.5 mL of PB pH 8 and 50 µL of Ellman’s reagent (4 mg/mL in PB pH 8.0) and stirred for 15 min at room temperature in the dark. Finally, these solutions were poured into a 96-well plate, and the absorbance was measured using a Synergy™ HTBioTek^®^ (BioTek Instruments, Winooski, VT, USA) at a wavelength of 412 nm. Absorbance readings were taken at time points of 0, 72, and 160 h. The results were analyzed and translated to concentrations using a standard calibration curve of L-cysteine in DDW containing Ellman’s reagent solution. The amount of free unreacted thiol groups was calculated from a mass balance.

### 2.9. Mechanical Characterization

Young’s modulus was determined from compression assays using a Lloyd mechanical testing machine (AMETEK, Berwyn, PA, USA). Samples were prepared as described in Section 2.5 and compressed at a rate of 1 mm/min, with a compressive displacement of up to 1.5 mm. Young’s modulus was calculated from the linear region of the stress–strain curve, typically up to 10% strain. Experiments were performed in quadruplicate.

### 2.10. Swelling

Swelling experiments were conducted to determine the swelling ability and the time required for swelling equilibrium. Kinetic experiments were performed in quadruplicate on hydrogels created from solutions with different pH values and after different curing times. After curing, the hydrogels were placed in a stainless steel grid submerged in a Petri dish containing 50 mL DDW at room temperature. To minimize water evaporation, the Petri dish was covered during the experiment. Each hydrogel was weighed periodically after wiping excess water with Kimwipes^®^ (Kimberly-Clark™, Roswell, GA, USA) and returned immediately to the Petri dish. The swelling percentage %*Q* at each time interval was determined gravimetrically and calculated as follows:

(1) $$\%Q=\;\frac{W_{t}-W_{0}\;}{W_{0}}\times 100\%$$

where *W*_0_ is the initial weight of the hydrogel, and *W_t_* is the weight of the hydrogel at time *t*.

The equilibrium swelling was calculated from the swelling vs. time curve, and the initial weight gain rate, r, defined as the slope, was calculated from the linear part of the curve representing the change in %*Q* with time. Swelling at equilibrium was estimated from the swelling vs. time curve for each type of hydrogel after 24 h of swelling.

### 2.11. Small-Angle X-ray Scattering (SAXS)

SAXS experiments were performed as previously described by Josef et al. [21] using a Molecular Metrology SAXS system equipped with a sealed microfocus tube (MicroMax −002 + S) emitting CuKα radiation. The scattering patterns were recorded by a two-dimensional position-sensitive wire detector (Gabriel). The scattered intensity, I(q), was recorded, where q is the scattering vector defined as q = 4sin(θ)/λ, 2θ is the scattering angle, and λ is the incident wavelength. After preparing the hydrogels, they were immediately poured into a thin-walled glass capillary (diameter of 2 mm and wall thickness of 0.01 mm) and sealed. SAXS measurements were recorded at time points of 0, 72, and 160 h.

### 2.12. Texture Profile Analysis (TPA)

The TPA of the hydrogels (height of 15 mm, diameter of 26 mm) was carried out using a Lloyd textile profile TA1 texture analyzer (AMETEK, Berwyn, PA, USA) equipped with a 10 N load cell. The analysis protocol consists of a two-cycle compression test to a maximum deformation of 50% between two parallel plates. The hydrogels were compressed at a rate of 30 mm/min at room temperature. The adhesive force, adhesiveness, and hardness were obtained from the texture profile and analyzed. Five samples were measured for each formulation.

### 2.13. Statistical Analysis

Statistical analysis was performed using Microsoft Excel software. Data from independent experiments were quantified and analyzed for each variable. Comparisons between multiple treatments were made with analysis of variance (ANOVA), and ad-hoc comparisons between two treatments were made using a two-tail Student’s *t*-test. A *p*-value of < 0.05 was considered statistically significant. Standard errors of the mean were calculated and presented for each treatment group.

## 3. Results and Discussion

### 3.1. Conditions of Hydrogel Formation

The hydrogels investigated in this study contained two polymers: acrylated chitosan and thiolated pectin. We anticipated that dual cross-linking could exist in this system: one arising from electrostatic interactions between the positively charged amine in chitosan and the negatively charged carboxylic acid on pectin, and the other from the chemical Michael-type addition reaction between thiol and acrylate. As the reaction kinetics between thiol and acrylates is known to be slow [17], the gelation process was studied over time.

**Figure 1:**
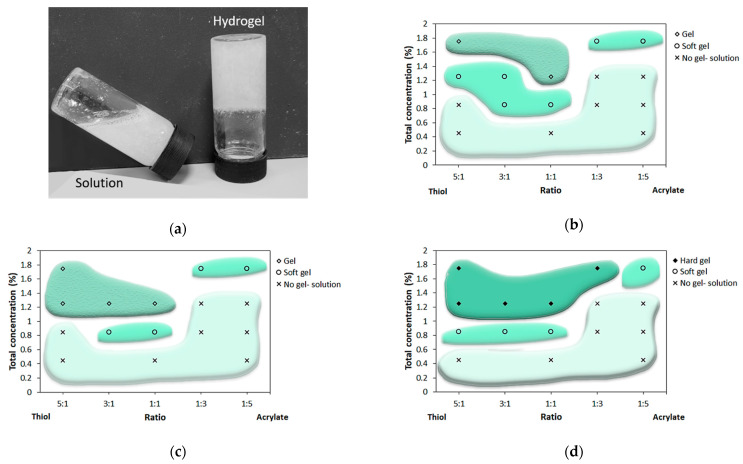
(**a**) The vial tilting method representing a “solution” phase and a “hard gel” phase. Phase diagram of the pectin-thiol and chitosan-acrylate hydrogels in a fixed pH value of 5.6 after curing for (**b**) 72, (**c**) 120, and (**d**) 160 h. *n* = 3.

To screen different formulations, we mixed a solution of thiolated pectin with a second solution of acrylated chitosan at room temperature and applied the tilt method to classify the mixture as a solution, a soft gel, a gel, or a hard gel (Figure 1). Different thiol-to-acrylate ratios and various total concentrations were studied, keeping the pH value constant using the acetate buffer at pH 5.6. This pH value was chosen following the results presented in a previous study by Marudova et al. [11], where the gelation of chitosan and pectin was studied. Chitosan was found to function as an effective crosslinker of pectin with a relatively low degree of esterification of 36%. The gelation process was initiated spontaneously upon mixing the two solutions. Raising the temperature to 37 °C had no effect on the curing time (data not shown). As shown in Figure 1b–d, mixtures with a total concentration lower than 0.4% did not form hydrogels, regardless of the thiol-to-acrylate ratio, probably because the concentration of the polymer chains was too low to create enough entanglements to form a three-dimensional matrix. This result did not change at longer gelation times. After 72 h of cross-linking, the gelation ability relied on both the thiol-to-acrylate ratio and the concentration. When the thiols were in excess, increasing the total concentration resulted in a transition from a solution to a soft gel (Figure 1b), whereas a further increase in concentration led to the formation of a hard gel. The solution to soft gel transition concentration and the soft gel to hard gel transition concentration seemed to decrease when the thiol excess decreased. Excess acrylate impaired the gelation ability, and the mixtures exhibited properties of a solution even when the total concentration was increased. Only at a high total concentration of 1.75% did the polymer mixture exhibit properties of a gel. Figure 1c shows the phase diagram after 120 h of curing and presents a similar trend compared with 72 h of curing. An exception was the formulations with a high thiol-to-acrylate ratio at a total concentration of 1.25%. These formulations were classified as soft gels after 72 h of curing but turned into gels after 120 h. This trend of soft gels, which became firmer and harder, was even more pronounced in the phase diagram for hydrogels that were left to cure for 160 h, as presented in Figure 1d.

Figure 1 also shows that hydrogels prepared with a high thiol-to-acrylate ratio transformed from soft gels to gels and hard gels with time, indicating that the cross-linking was a slow process. These findings are consistent with those of previous studies on poly (ethylene glycol) (PEG)-based hydrogels formed using the Michael-type addition reaction between thiol and acrylate. This reaction has a step-growth polymerization mechanism, which is known to have a slow polymerization rate [17,22].

### 3.2. Interaction between Acrylated Chitosan and Thiolated Pectin

Following the results presented in the previous section, a ratio of 5:1 thiol to acrylate and a total concentration of 1.25% were chosen for further investigations. This formulation seems to be the most interesting since it showed intermediate properties of a soft gel 72 h after curing, and further displayed evolution with time from soft gels to gels and finally to hard gels. Thus, we investigated the changes in the properties depending on the curing time. The effect of pH, which is known to play a key role in polyelectrolyte complex formation, was also analyzed because it affects the degree of ionization of the functional groups.

#### 3.2.1. Electrostatic Interactions

**Figure 2:**
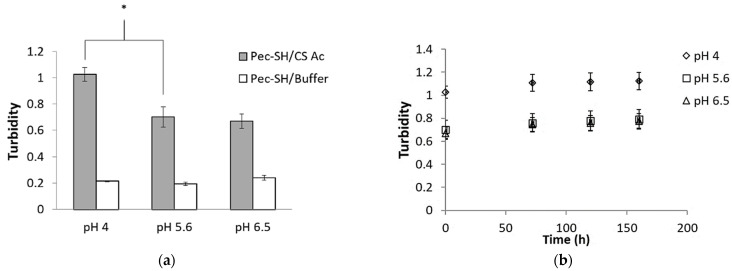
Turbidity measurements of hydrogels prepared at different pH values. (**a**) Immediately after mixing and casting. (**b**) Change with time. The bars represent the standard error of the mean, *n* = 3. (*) refers to a statistically significant difference at *p* < 0.05.

Electrostatic interactions between the carboxylic acid of pectin and the amine group of chitosan can be generated at pH values between 3 and 6, which are above the pKa of pectin but below the pKa of chitosan [11,23,24]. However, in this hybrid system, the bulky side groups of PEGDA carried by chitosan could interfere with the complex formation. Therefore, we were interested in charactering the extent of electrostatic interactions in the hydrogel matrix. The use of turbidity measurements allowed for the examination of the extent of the polyelectrolyte complexation. A similar methodology was previously used to evaluate the magnitude of electrostatic interactions between cationized gelatin and gum arabic [25] and the formation of gelatin and the k-carrageenan complex [26]. Figure 2a shows that the hydrogels prepared from thiolated pectin and acrylated chitosan had high turbidity values compared with the transparent control solutions made of only thiolated pectin and buffer. The hydrogels prepared at pH 4 had the highest turbidity value (Figure 2a). Furthermore, the turbidity of thiolated pectin and acrylated chitosan hydrogels was found to be significantly pH dependent (ANOVA, *p* < 0.05), and that of the control was almost constant. Turbidity was also examined at different gelation times, but its value was constant with time (ANOVA, *p* > 0.5, Figure 2b).

The high turbidity at pH 4 is attributed to the complex formation. This indicates that the strong electrostatic interactions between the negatively charged carboxylate groups of pectin and the positively charged amino groups of chitosan [5,27] are not hampered due to acrylation or thiolation. The increase in pH causes deprotonation of the amine groups present in chitosan, resulting in a reduction of the net charge in the gel. This eventually leads to depletion in the pectin–chitosan interactions. Higher pH values promote other reactions, such as the Michael-type addition and formation of disulfide bonds [14,28]. The constant turbidity with time suggests that the rate of reaction in the case of electrostatic interactions is very fast, causing turbidity to form instantly. Moreover, it shows that other interactions that participate in hydrogel formation do not affect turbidity values.

#### 3.2.2. Chemical Reaction

**Figure 3:**
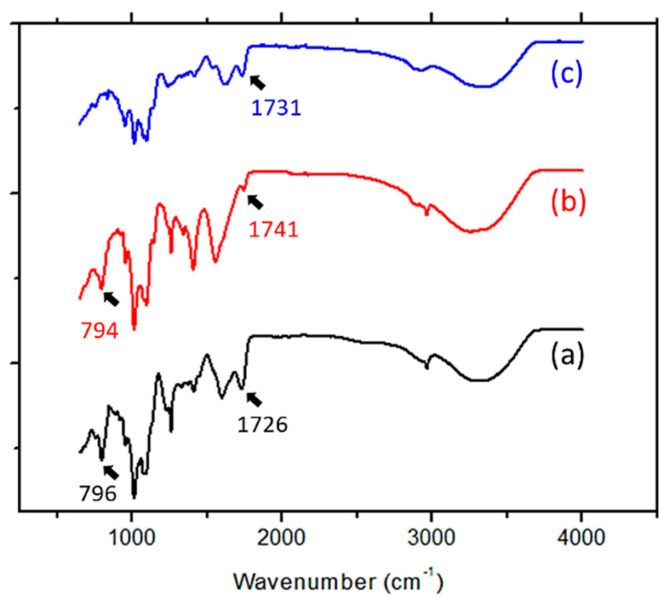
FTIR analysis of hydrogels prepared at (**a**) pH 4, (**b**) pH 5.6 and (**c**) pH 6.5.

The Michael-type addition reaction between pectin thiol and chitosan acrylate was verified by analyzing the FTIR spectra. The FTIR spectra of the hydrogels obtained after 72 h of cross-linking at different pH values are shown in Figure 3. Two typical peaks attributed to the presence of PEGDA are seen between 1800 and 1600 cm^−1^ (stretching vibration of ester) and 800 cm^−1^ (stretching vibration of C=C) [29].

The spectra from the hydrogels prepared at pH 4 and pH 5.6 presented two typical peaks, indicating that some acrylate groups remained unattached. However, in the spectra of the hydrogels prepared at pH 6.5, the stretching vibration peak of the C=C group at 800 cm^−1^ disappeared. These observations suggest that the Michael-type addition reaction at low pH was not fully completed, whereas the acrylate groups were consumed by this reaction at a high pH value of 6.5 after 72 h.

**Figure 4:**
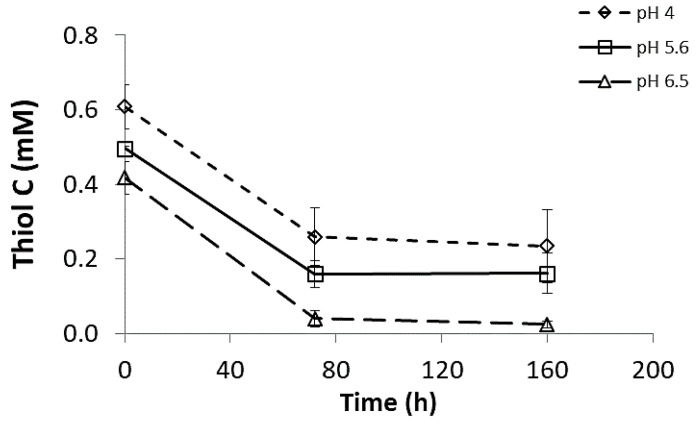
Kinetic analysis was performed using Ellman’s reagent representing the thiol concentration in gel vs. time. The bars represent the standard error of the mean, *n* = 3.

To better understand the reaction mechanism, a kinetic study was performed by monitoring the amount of free thiols in the hydrogels at predetermined gelation times [30]. As shown in Figure 4, the initial concentration of free thiols was low for pH 6.5 compared with other pH values. At this pH value, disulfide bonds are typically generated. Yom-Tov et al. showed that the extent of disulfide bond formation could affect the hydrogel properties in a cross-linked hydrogel system composed of PEG-4SH and PEGDA [17]. They demonstrated that a network of tetra-PEG is obtained by forming disulfide bonds in a solution upon mixing. Since the polymerization reaction had already been started during the reagent’s separate mixing, the formation of the final network resulted in a faster gelation rate once PEG-4SH networks were combined with PEGDA solutions. Pectin is a highly branched polysaccharide capable of self-gelation at acidic pH [31]. The addition of thiol groups to this polymer allows for self-gelation at high pH values via disulfide bridges [28]. Thus, the data presented in Figure 4 imply that, at a high pH value, a preliminary network composed of disulfide bonds is created during the reagents’ separate mixing phase. Taking these results together with the ones in Figure 1, we hypothesize that a preliminary network must be formed to create a stable gel. Therefore, when the thiol-to-acrylate ratio is low, a gel state is not formed, probably because there are not enough thiols to create the preliminary network. The free thiol concentration decreased after 72 h of cross-linking for all the examined pH values and reached a plateau, as it was not changed further statistically after 160 h (*p* > 0.5, Figure 4). These results support the claim that, in this hydrogel system, the Michael-type addition reaction is very slow.

### 3.3. Mechanical Characterization

**Figure 5:**
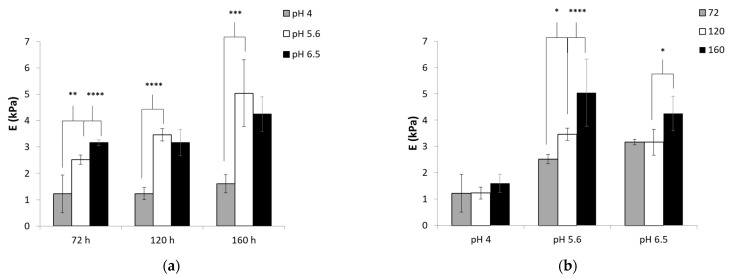
Young’s modulus of the pectin thiol and chitosan acrylate hydrogels (**a**) vs. gelation time and (**b**) vs. pH value during preparation. The bars represent the standard error of the mean, *n* = 5. (*) refers to a statistically significant difference at *p* < 0.05, (**) refers to a statistically significant difference at *p* < 0.01, (***) refers to a statistically significant difference at *p* < 0.005, and (****) refers to a statistically significant difference at *p* < 0.0001.

The stiffness of the hydrogels, described as Young’s modulus, was evaluated as a function of time and pH value (Figure 5). An increase in the modulus as a function of the pH was observed for all the examined gelation periods. After 72 h of cross-linking, increasing the pH from 4 to 5.6 resulted in a significant increase in the modulus from 1.2 to 2.5 kPa (*p* < 0.01). A further increase in pH from 5.6 to 6.5 caused a further increase in the modulus from 2.5 to 3.2 kPa (*p* < 0.0001). After 120 h of cross-linking, an increase in the modulus value was detected when the pH value increased from 4 to 5.6 (*p* < 0.0001). However, a further increase in modulus was not obtained after a further increase to pH 6.5. A similar trend was observed in a longer gelation period of 160 h (*p* < 0.005). Figure 5b shows the relationship between the modulus and the gelation time. At pH 4, the modulus was constant and did not change with the increase in gelation time. However, at higher pH values, the modulus increased when the gelation was allowed to proceed for longer times.

As the chemical composition and the polymer concentration are identical for all studied samples, the stiffness of the hydrogels was determined by their degree of cross-linking. Electrostatic interactions lead to a complex formation, which acts as physical cross-links. These interactions were formed between the positive amine group of acrylated chitosan and the negative carboxylic acid of thiolated pectin. Our turbidity measurements demonstrated that the magnitude of the electrostatic interactions was favorable at pH 4 and diminished at pH 5.6 and 6.5. The covalent bonds were established by the Michael-type addition reaction between the acrylate conjugated to chitosan and the thiol group in thiolated pectin. This thiol–ene click chemistry was triggered by the thiolate anion, which acts as a nucleophile and attacks the carbon of the alkene [14,16]. The number of thiolate anions also depends on the pH of the solution; the reaction is favorable when the pH values are high [32]. Our FTIR and thiol content results show that the extent of chemical reaction increased with pH. Thus, as the pH increased, electrostatic interactions became less prominent, whereas the extent of chemical links was enhanced. Therefore, the strengthening of the mechanical characteristics with the increase in pH indicates that covalent bonds are more significant than electrostatic interactions in determining the stiffness of the hydrogels.

In comparing the results after different gelation periods, the stiffness increased at times longer than 72 h only at pH values of 5.6 and 6.5, suggesting that the cross-linking density increased. As was mentioned earlier, previous studies have shown that the Michael-type addition reaction between thiol and acrylate has slow kinetics [15]. Yom-Tov et al. evaluated the mechanical characteristics of PEG-thiol and PEGDA cross-linked hydrogels with a curing time of up to 7 days [17]. Our observations support this conclusion, indicating that a slow gelation mechanism is involved mainly at high pH values.

To conclude, by extending the gelation time and/or the pH value of the polymers, the reaction conversion may be improved, and better mechanical properties can be obtained.

The results in this section demonstrate that, for the studied samples, the properties of hydrogels changed significantly between curing times of 72 and 160 h. Therefore, these curing times were chosen for further examination.

### 3.4. Effect of pH on Swelling Capacity

**Figure 6:**
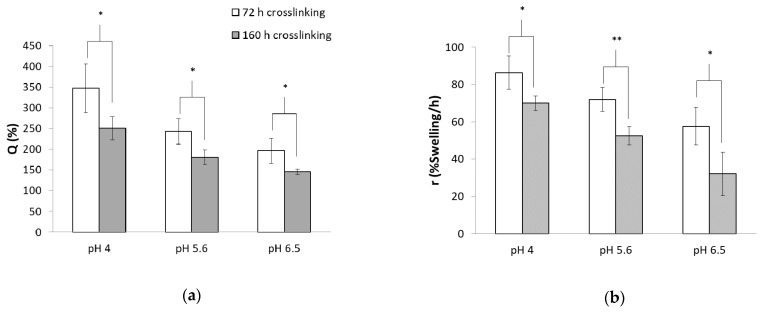

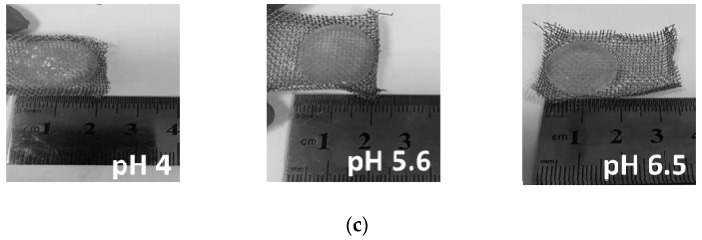
(**a**) Q-Swelling in equilibrium and (**b**) initial water gain rate vs. pH value during preparation. (**c**) Swollen hydrogels cross-linked for 160 h. The bars represent the standard error of the mean, *n* = 4. (*) refers to a statistically significant difference of *p* < 0.05, and (**) refers to a statistically significant difference of *p* < 0.01.

As swelling ability is one of the most important characteristics of hydrogels, the effect of pH value and gelation time on swelling was examined (Figure 6). An increase in curing time from 72 h to 160 h led to a significant decrease in swelling ability for all the examined pH values (Figure 6a). The hydrogels prepared at pH 4 showed an equilibrium swelling of 350% after 72 h of cross-linking and after 160 h they displayed a swelling ability of 250% (*p* < 0.05). The same trend was observed for pH 5.6 and 6.5 in which the equilibrium swelling decreased from 240% to 180% (*p* < 0.05) and from 190% to 140% (*p* < 0.05), respectively. The water uptake of the hydrogels in equilibrium was found to be significantly pH dependent for both of the examined gelation periods (ANOVA, 72 h *p* < 0.01, *n* > 3, 160 h *p* < 0.0005, *n* > 3).

Figure 6b illustrates the swelling rate of the hydrogels calculated from the slope of the linear part of the swelling vs. time curves. Hydrogels cured for 72 h swelled faster than those cured for 160 h. The rate of swelling was found to be significantly pH dependent (ANOVA, 72 h *p* < 0.05, *n* > 3, 160 h *p* < 0.0005, *n* > 3), indicating that as the pH value increased, the rate of swelling decreased.

Figure 6c demonstrates that hydrogels fabricated at various pH values and cross-linked for 160 h differ in their swelling ability.

The greater swelling ability and faster swelling rate of hydrogels cured for a shorter gelation time indicate a lower cross-linking density [17]. Further, an inverse correlation between Young’s modulus values and swelling behavior was well established in previous studies [33].

The increase in pH affected the swelling behavior, consistent with our previous suggestion that as the pH increases, the Michael-type addition reaction becomes more favorable. A high reaction conversion increased the cross-linking density, which decreased the swelling ability and swelling rate.

### 3.5. Nanostructure Examination Using SAXS

The inhomogeneities in the hydrogels led us to hypothesize that the nanometric structure could change with time and pH. Thus, we gathered information related to the hydrogel structure using SAXS.

**Figure 7:**
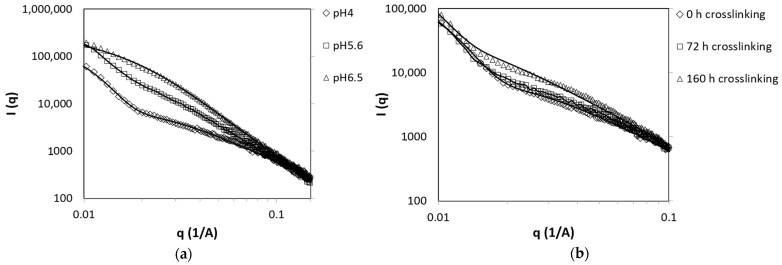
SAXS curves of hydrogels (**a**) at different pH values immediately after mixing and (**b**) at pH 4 at different curing times. Lines represent the fit to the aggregate and network model (Equation (2)) for pH 5.6 and pH 6.5, and Equation (3) for pH 4.

The SAXS patterns of hydrogels at different pH values immediately after mixing are presented in Figure 7a. The results show no apparent difference at large scattering angles as the plots overlap. At smaller scattering angles, the curves became sensitive to the pH of the hydrogel. The change in the shape of the curve, particularly the appearance of a shoulder at the intermediate pH value, suggests that the scattering curve reflects a superposition of two contributions: one arising from the polymer network that dominates mainly at high scattering angles and the other from the formation of aggregates that dominate at small angles.

We attempted to fit the SAXS plots to two models describing inhomogeneous hydrogels. The first model is the classical Ornstein–Zernike model with a Debye–Bueche term, which is commonly used to describe hydrogels [34,35]. The form factor *P*(*q*) is represented by

(2) $$P\left(q\right)=\;\left(\frac{k_{net}\;}{1+{\left(q\xi_{net}\right)}^{2}}\right)+\left(\frac{k_{agg}\;}{{\left(1+{\left(q\xi_{agg}\right)}^{2}\right)}^{2}}\right)$$

where *ξ_net_* is the correlation length of the network, *ξ_agg_* is the dimension of aggregates, *k_net_* is the constant of the network, and *k_agg_* is the constant of the aggregates.

The second model was previously used to describe inhomogeneous polyacrylamide hydrogels [36].

(3) $$P\left(q\right)=\;\left(\frac{k_{net}\;}{{\left(1+{\left(q\xi_{net}\right)}^{2}\right)}^{1/2}}\right)+k_{agg}\exp\left(-\frac{1\;}{3}+{\left(qR_{agg}\right)}^{2}\right)$$

where *ξ_net_* is the correlation length of the network, *R_agg_* is the dimension of aggregates, *k_net_* is the constant of the network, and *k_agg_* is the constant of the aggregates.

Equation (2) shows a good fit to the scattering from hydrogels prepared at pH 6.5 and as well as pH 5.6, while a better fit for pH 4 was obtained with Equation (3). The parameters calculated from the fitting are presented in Table 1. The results suggest that the increase in the degree of inhomogeneity with pH was caused by the increase in the number of aggregates. The increase in mesh size with pH can also be attributed to enhanced aggregation. Fewer chains are available for the creation of the network as they form the aggregates. The aggregates contribute to the cross-linked network, and thus the swelling degree decreases as the curing time increases (Figure 6a).

Figure 7b shows the change in the scattering curve with the increase in curing time for pH 4. At this pH, the hydrogels presented an increase in the mesh size of the net (*ξ_net_*) with time (Table 1). The characteristic size of the aggregates, *R_agg_*, also increased simultaneously. The parameter *k_agg_*, which represents the amount of aggregates, increased with time, while the parameter *k_net_* decreased (Table 1). Thus, similar to the effect of pH, increasing the curing time at pH 4 led to aggregation. For hydrogels prepared at pH 5.6 and pH 6.5, the scattering curve did not change with time (data not shown).

We can conclude that the structure of hydrogels may be inhomogeneous and composed of a gel matrix with a mesh size that decreases over time and heterogeneous clustered regions that may function as cross-linking spots. This increase in heterogeneous regions may be a result of the Michael-type addition reaction at a long curing time, which is less favorable at a low pH value.

### 3.6. TPA Measurements

While performing the experiments, we observed that the hydrogels adhered to different surfaces. To quantify the adhesiveness, we used texture analysis, which is a common technique employed in the industry for the mechanical characterization and evaluation of food textural behavior [37]. It was previously used as a method for pharmaceutical gel characterization [38].

**Figure 8:**
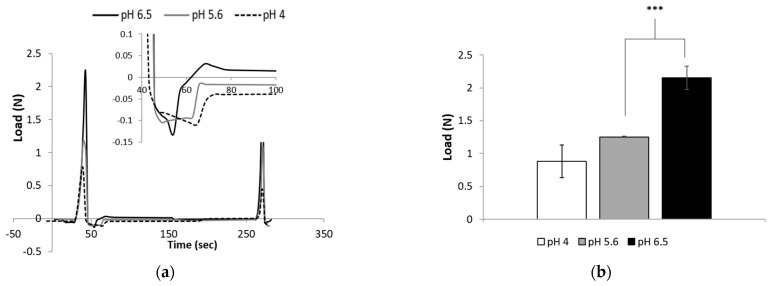

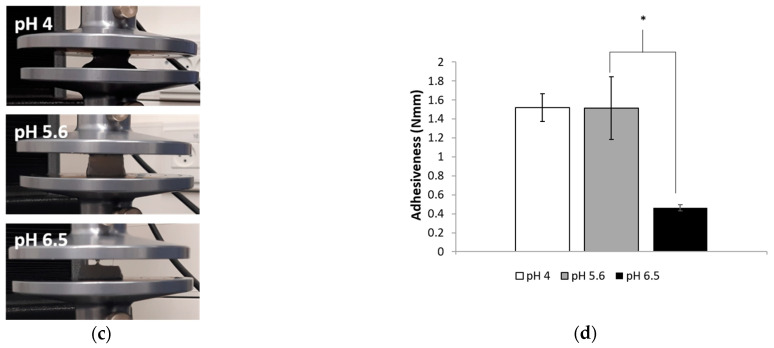
Texture profile analysis experiment. (**a**) Force–deformation curves, (**b**) hardness (ANOVA *p* < 0.005), (**c**) qualitative observation of the adhesiveness, and (**d**) adhesiveness (ANOVA, *p* < 0.05) of hydrogels cross-linked for 160 h and prepared at three different pH values. The bars represent the standard error of the mean, *n* = 5. (*) refers to a statistically significant difference of *p* < 0.05, and (***) refers to a statistically significant difference of *p* < 0.005.

Figure 8a illustrates the force–deformation curves of hydrogels cross-linked for 160 h and prepared at three different pH values. After the first compression, the three hydrogels presented a negative area associated with the adhesiveness property of the gel (Figure 8a). The hardness of the hydrogels increased significantly with the increase in pH from 0.88 ± 0.25 N for pH 4 to 2.15 ± 0.39 N for pH 6.5 (ANOVA, *p* < 0.005, Figure 8b).

A large difference was observed qualitatively between hydrogels prepared at different pH values and similar curing times (Figure 8c). The hydrogels prepared at pH values under the pKa of chitosan were sticky and adhered to the upper plate, whereas those prepared at pH 6.5 were not and did not. Figure 8c shows the adhesiveness of the hydrogels measured as the negative work between the two cycles. The results support the qualitative observation: the adhesiveness of the hydrogels was high at low pH values and decreased statistically at pH 6.5 (ANOVA, *p* < 0.05, Figure 8d).

Figure 8 shows that high adhesiveness is associated with hydrogels prepared at low pH values. Our previous experiments revealed that, under these conditions, the thiol concentration in the hydrogel is high. Unattached thiol groups are known to present adhesive properties that are exploited in many fields, such as semiconductors [39], sticky surfaces [40,41], and bioadhesion [42]. This chemical group can bind different molecules. For example, in the field of mucoadhesion, thiols can form disulfide bonds with glycoproteins, which are secreted by the mucosal tissue. Therefore, it is reasonable to assume that the free thiol groups on the hydrogel surface are responsible for the adhesion properties. Hydrogels prepared using a pH 6.5 buffer presented a low concentration of free thiols, thus showing low adhesiveness in the TPA assay (Figure 8c,d).

The upper plate of the texture analyzer device is composed of aluminum, which is also known to bind thiols [43].

The adhesive properties of the novel acrylated chitosan/thiolated pectin hydrogels may open the possibility of their use as mucosal mimetic surfaces. Mucoadhesion is the term describing the ability of a material to adhere to mucosal surfaces [44]. This field has attracted much attention in the past few decades, as mucoadhesive materials increase the residence time of drugs at the application site [45,46,47]. Early stages in the development of novel mucoadhesive systems require the utilization of animal tissues for adhesion testing. However, experiments performed on ex vivo tissues may lead to unreliable results due to technical limitations, such as the large variance between animals. In addition, the will to avoid animal killing raises the need for alternative substrates. Recently, several synthetic model surfaces have been developed in an attempt to mimic mucus layer properties [48,49,50]. Thiolated pectin hydrogels were previously studied by Dozli et al., who claimed that the hydrogels combine all the main features of the mucosa (gel-like character, negative charge, sugar moieties and free thiol groups) in one substrate [51]. They explained that this may increase the similarity to the mucosa and thus improved the substrate’s performance. Another feature not discussed in the literature with respect to mucosal mimetic surfaces is the pH. Different mucosal membranes in the body have different pH values; for example, the pH in the vagina is 4 [52], while its value in the nose or intestine is between 5.5 and 6.5 [53]. The pH of the mucosal surface is crucial for the accurate examination of mucoadhesive formulations. Therefore, the hydrogel system presented in this research, which is similar to the one described by Dozli et al., could demonstrate another characteristic in its physical resemblance to natural mucosal tissue. Further studies could confirm that this hydrogel system could be used as a mimetic, with high similarity to the mucosa of various regions in the body.

## 4. Conclusions

A novel hydrogel system based on the physical and chemical interactions between thiolated pectin and acrylated chitosan was developed and characterized. This hybrid system was found to show different properties when gelation was conducted at different pH values and curing times. Turbidity measurements revealed that at low pH values below the pKa of chitosan, more electrostatic interactions were formed between opposite charges. However, at high pH values, the FTIR results showed the occurrence of the Michael-type addition reaction between the acrylate and thiol. Mechanical characterization demonstrated that increasing the pH value created stiffer hydrogels. SAXS measurements revealed that the nanostructure of the hydrogels was inhomogeneous and composed of a gel matrix with a mesh size that decreases over time and heterogeneous clustered regions, which could function as cross-linking spots. The texture profile analysis assay showed that hydrogels prepared at a pH below the pKa of chitosan had greater adhesiveness, and this property was attributed to the free thiol groups in the gel. The hybrid hydrogel system has controllable properties and can be applied to develop tailor-made biomaterials for specific applications. It could be a good candidate for a wide range of biomedical applications, such as substrates for mucosa-mimetic materials, which have a greater resemblance to the natural mucosal tissue of various regions in the body.

## Figures and Tables

**Table 1:** Parameters derived from fitting the scattering curves of hydrogels at different pH values and curing times.

	Time [h]	*ξ_net_* [Å]	*R_agg_* [Å]	*k_net_*	*k_agg_*
pH 4	0	38.2	208.4	9314.7	225,747.1
	72	41.4	225.0	12,193.3	269,394.3
	160	87.7	274.7	57,456.9	632,203.7
pH 5.6	0	265.8	99.8	538,136.6	291,854.0
	72				
	160				254,269.9
pH 6.5	0	327.1	47.8	521,801.3	184,615.5
	72				
	160				237,117.1

## Data Availability

The data presented in this study are available on request from the corresponding author.
